# A GIS and field-based assessment of the ecological consequences of illegal mining (galamsey) on blackfly breeding sites in Ghana: implications for the sustainable development goals

**DOI:** 10.1186/s41182-026-00944-4

**Published:** 2026-03-31

**Authors:** Jeffrey Gabriel Sumboh, Gabriel Appiah, Frank Teye Oblim, Emmanuel Oboubie, Michael David Wilson, Satoshi Kaneko, Dziedzom K. deSouza

**Affiliations:** 1 Department of Parasitology, Noguchi Memorial Institute for Medical Research, University of Ghana, Legon, P.O. Box LG 581, Accra, Ghana; 2 Graduate School of Biomedical Sciences, Nagasaki University, 1-12-4 Sakamoto, Nagasaki, 852-8523 Japan; 3 Department of Eco-Epidemiology, Institute of Tropical Medicine (NEKKEN), Nagasaki University, 1-12-4 Sakamoto, Nagasaki, 852-8523 Japan; 4 Water Research Institute, Council for Scientific and Industrial Research, P. O. Box AH 38, Achimota, Accra Ghana

**Keywords:** Blackflies, Illegal mining, Vector ecology, Land cover change, Onchocerciasis, Remote sensing

## Abstract

**Background:**

The Ofin River basin has historically supported *Simulium* blackfly breeding, vectors of *Onchocerca*, yet recent Programme reports have noted a sharp decline in monitored populations. With illegal artisanal mining (galamsey) expanding around the basin, this study assessed how associated ecological changes may influence habitat suitability for blackfly breeding across three riverine communities (Adwuman, Buabenso, and Kyekyewere).

**Methods:**

Water discharge and quality were assessed through field measurements and laboratory analyses of pH, electrical conductivity, turbidity, colour, total suspended solids (TSS), and total dissolved solids (TDS). Satellite imagery from 2008, 2017, and 2022/23 was analysed using Iso Cluster Unsupervised Classification algorithm (ISODATA), Principal Raster Components Analysis (PRCA) and the Normalized Difference Vegetation Index (NDVI) to quantify transitions and vegetation health using ArcGIS Pro.

**Results:**

Water discharge rates varied (Adwuman: 181.57 m^3^/s, Buabenso: 78.93 m^3^/s, Kyekyewere: 111.95 m^3^/s) and quality analysis showed differences in key parameters. Adwuman’s pH was 6.98, conductivity (145.5 µS/cm), turbidity (3392.5 NTU), colour (3375 Hz) and TSS (3630 mg/L). Buabenso had a pH of 6.98, conductivity 146.75 µS/cm, turbidity 3525 NTU, colour at 3812.5 Hz and TSS of 3857.5 mg/L. Kyekyewere recorded the lowest pH (6.95) and conductivity (145.25 µS/cm), but the highest turbidity (3725 NTU), colour (4175 Hz) and TSS (4342.5 mg/L). Forest cover declined by 10.72, 7.41, and 8.80 percentage points in Adwuman, Buabenso, and Kyekyewere, respectively, while light vegetation increased by 15.71, 15.00, and 18.93 points. Water coverage expanded by 10.81, 6.12, and 5.26 percentage points across the communities, indicating hydrological alteration. NDVI revealed widespread declines in vegetation health and density, particularly near mining zones.

**Conclusion:**

The combined effects of extreme sedimentation, vegetation degradation and riparian disturbance suggest ecological conditions that are increasingly unsuitable for blackfly breeding in the Ofin River basin. The disruptions also threaten food security, clean water access and ecosystem integrity, with implications for achieving SDG 2, 3, 6, and 15. Strengthened River management, reforestation of degraded riparian areas, enforcement against illegal mining and community-based monitoring are needed to restore ecological function and safeguard both biodiversity and public health.

**Supplementary Information:**

The online version contains supplementary material available at 10.1186/s41182-026-00944-4.

## Introduction

Illegal and unregulated small-scale alluvial mining has emerged as a major ecological threat across many tropical regions, driving river degradation, deforestation and sedimentation that disrupts freshwater ecosystems [1–3]. In sub-Saharan Africa and particularly Ghana, Artisanal and Small-scale Mining (ASM) activities, locally termed galamsey have intensified raising concerns about their impacts on water quality, food security and ecosystem integrity [4]. These ecological disturbances alter river morphology, increase sediment loads and degrade riparian vegetation with cascading effects on biodiversity and community livelihoods [5]. One critical but overlooked consequence is the disruption of habitats suitable for *Simulium damnosum sl*, the blackfly vector that breeds in fast-flowing and oxygen-rich rivers [6, 7].

Blackflies serve as the vectors of *Onchocerca volvulus*, the filarial parasite responsible for human onchocerciasis [8]. Globally an estimated 249.5 million people across 28 endemic countries remain at risk of infections [9] with 18.8 million people requiring preventive chemotherapy (PC), 99% of which are in sub-Saharan Africa including Ghana [10]. The disease leads to chronic dermatological and ocular morbidity, including severe disabling itching and vision impairment [11]. Larval development requires substrates such as vegetation or stones to provide attachment surfaces [12]. Blackfly abundance typically fluctuates with seasonal river dynamics, increasing periods of flow when suitable breeding substrates are abundant.

Illegal mining activities, particularly the excavation and churning of riverbeds disrupt natural flow regimes, increase turbidity and modify the substrates required for blackfly larval attachment [13]. The expansion of these activities is driven in part by local livelihoods pressures which has intensified human disturbance along river corridors [14]. These ecological disturbances have been associated with marked reductions in blackfly breeding activity in historically endemic areas, creating a complex paradox in which severe environmental degradation coincides with reduced vector presence and potentially lower transmission risk.

Long-term entomological monitoring along the Ofin and Pra rivers have documented a marked decline in *Simulium* breeding activity from the early 2000s onward, with several historically productive sites disappearing as illegal mining intensity increased [15]. Subsequent surveys including those by Lamberton et al. similarly reported the absence of blackflies in the lower Pra River communities attributing this pattern to severe riverine disturbances from gold mining [16]. These studies highlighted extreme turbidity and suspended sediment loads, conditions associated with incompatible blackfly larval development [17]. Recent surveillance by Ghana’s Neglected Tropical Disease (NTD) Elimination Programme similarly recorded little to no blackfly activity at formerly active monitoring sites within mining zones (unpublished programme data, 2022). Collectively, these entomological studies provide important historical context for understanding how ecological degradation has altered vector presence in the Ofin-Pra basin [16–18].

Although reduced vector presence may temporarily lower local transmission risk, such ecological disruption may also shift breeding activity to new or less monitored [14, 19] zones. The ecological and public health shifts intersect with broader development priorities, undermining progress toward some Sustainable Development Goals (SDGs) including SDG 3 (Good Health), SDG 6 (Clean Water), SDG 15 (Ecosystem protection) and SDG 2 (Food security) [20–22].

These observations motivated the present study which aimed to assess ecological degradation in three communities (Adwuman, Buabenso, and Kyekyewere) along the Ofin River, an area currently impacted by mining and historically known for blackfly breeding, to quantify changes in vegetation cover and riverine conditions associated with mining activities.

## Materials and methods

### Study area

**Fig. 1:**
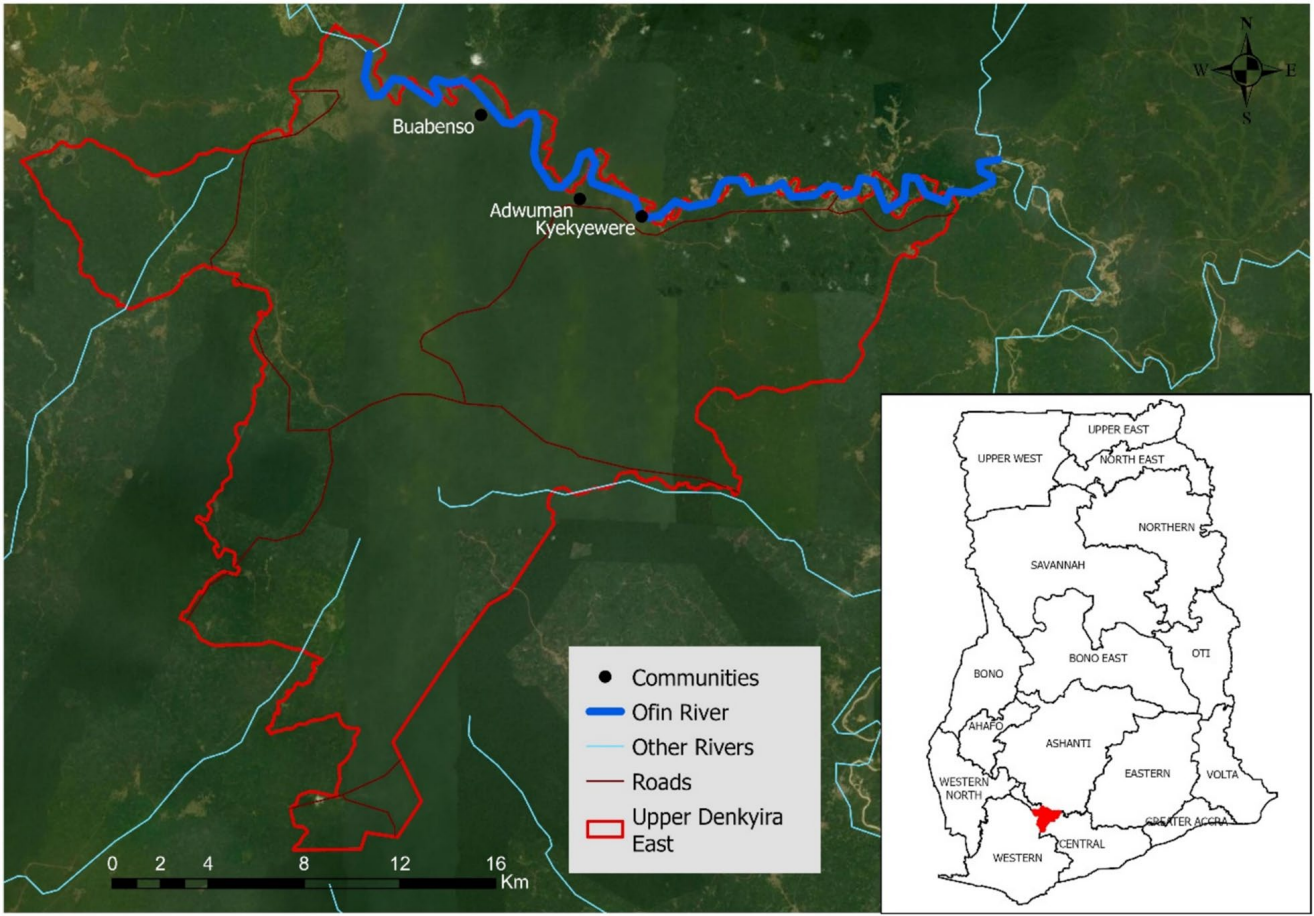
Location of communities and Ofin River within the study district

The study was conducted in the Upper Denkyira East Municipality of Ghana’s Central Region (Fig. 1), an area spanning approximately 1,020 km^2^ and bordered by five adjoining districts. The municipality lies within the semi equatorial climatic zone, with mean annual temperatures of 24–29 °C and annual rainfall ranging from 1,200 to 2,000 mm. The landscape is characterized by semi deciduous forest interspersed with agricultural land. Although agriculture remains the primary livelihood activity, the rapid expansion of artisanal and small-scale mining (ASM) has increasingly altered land use patterns and intensified human disturbance along the Ofin River.

### Study communities

Three riverine communities [Buabenso (−1.737845W, 5.956545N), Adwuman (−1.7007615W, 5.925026N) and Kyekyewere (−1.677546W, 5.918505N)] are situated along the Ofin River within the Upper Denykira East Municipality (Fig. 1) (Supplementary Figure S1). These communities were selected because they lie within historically documented blackfly breeding zones and have experienced extensive ecological disturbances from illegal mining activities [17]. Also, the NTD control Programme monitors blackfly populations within the Ofin river basin.

### River flow measurements and computation

Field measurements and water samples for laboratory analysis were collected at designated points within each of the three study communities. Sampling was conducted twice daily, once in the morning (before 12 noon) and once in the late afternoon/evening (after 3 pm) to capture potential diurnal variations in river flow and water quality. The river discharge measurement was done using the Valeport Suspended impeller current meters (models BM001 and BM002) applying the velocity-area method for open channel flow in accordance with ISO 748:2021 guidelines [23]. The discharge was computed using the standard velocity-area relationship shown in Eq. 1.

1 $$Q\, = \,V\, \times \,A$$

where: Q is the discharge (m^3^/s), V is the average flow velocity (m/s), A is the cross-sectional area of the portion of the channel occupied by the flow (m^2^).

### Physico-chemical parameters

Water quality was assessed using a combination of in-situ measurements and laboratory-based analysis to capture both immediate field conditions and validated reference values for blackfly survival. In-situ measurements were taken using a calibrated portable multiparameter water quality probe, which recorded temperature, pH, and total dissolved solids (TDS) at each sampling point. Additionally, twelve grab samples (four per community) were collected in pre-rinsed polyethylene bottles, stored on ice and transported to the laboratories of the Council for Scientific and Industrial Research (CSIR)-Water Research Institute for laboratory analysis. The laboratory analysis quantified, pH, conductivity, total suspended solids (TSS), colour and turbidity using standard methods for quality assessments [24]. Laboratory pH measurements were included to validate field readings and ensure analytical accuracy.

### Vegetation health analysis

To assess vegetation cover change and broader environmental degradation associated with illegal mining, Landsat imagery was obtained from the United States Geological Survey (USGS) archive and supplemented with high-resolution visual interpretation from Google Earth. All spatial analyses were conducted in ArcGIS 10.8 using standard remote-sensing workflows, including the computation of multiple vegetation indices to quantify vegetation health and land-cover change. An Iso Cluster Unsupervised Classification (ISODATA) algorithm was applied to group spectrally similar pixels and delineate dominant land cover classes prior to interpretation, providing an objective basis for identifying vegetation loss and mining related disturbance. A Vegetation Loss Index (VLI) was derived using Principal Raster Component Analysis (PRCA), which reduces dimensionality and enhances spectral contrast across image bands, enabling clearer detection of vegetation decline over time. ISODATA and PRCA analyses were performed on Landsat imagery from 2008, 2017, and 2022/2023 to characterize temporal changes in major land‑cover categories, including forest, light vegetation, built‑up areas, bare soil and water bodies. Although PRCA effectively distinguishes vegetation and water features, it may underrepresent built up areas in mixed use or mining affected zones where spectral signatures overlap with bare soil or disturbed substrates.

Additionally, The Normalized Difference Vegetation Index (NDVI) was also computed from the same satellite imagery using the standard formula;

$$NDVI = \frac{{\left( {Near\, Infrared\, Red\, band - Red\, band} \right)}}{{\left( {Near\, Infrared\, Red\, band + Red\, band} \right)}}$$

to quantify vegetation greenness and density. NDVI values range from − 1 to + 1, with higher values indicating healthier vegetation and lower values reflecting sparse or absent vegetation.

### Data processing and analysis

Water quality measurements from field and laboratory assessments were compiled in Microsoft Excel and summarized using descriptive statistics. Morning and afternoon values were compared descriptively to observe any diurnal differences. River discharge was calculated from measured velocity and its cross-sectional area. Because the study objective was descriptive and focused on comparing measured values with established ecological thresholds for *Simulium* breeding, no inferential statistical tests or p values were required.

For the spatial component, outputs generated from the remote sensing procedures (ISODATA classification, NDVI, and Vegetation Loss Index) were compared descriptively across the three time periods to identify proportional changes in land cover categories and visually assess vegetation loss and mining related disturbance.

## Results

### Community river discharge and in-situ water quality measurements

In-situ measurements (Table 1) showed average river discharge values of 181.57 m^3^/s at Adwuman, 78.93 m^3^/s at Buabenso, and 111.95 m^3^/s at Kyekyewere. Field water quality measurements indicated slight variation in pH, total dissolved solids (TDS), and temperature across the three communities. At Adwuman, pH measured 6.87, with TDS of 154.85 mg/L, and temperature of 27.80 °C. At Buabenso, the mean pH was 6.82, with TDS of 158.33 mg/L and temperature of 27.53 °C. Kyekyewere recorded a pH of 7.02, TDS of 155.73 mg/L, and temperature of 27.50 °C.

**Table 1:** In-situ river discharge and field water quality measurements

Parameter	Adwuman	Buabenso	Kyekyewere
River discharge (m^3^/s)	181.57	78.93	111.95
pH	6.87	6.82	7.02
TDS (mg/L)	154.85	158.33	155.73
Temperature (°C)	27.8	27.53	27.5

Laboratory analysis showed slight site-specific variation in water quality parameters across the three communities (Table 2). Adwuman recorded a similar pH of 6.98, with slightly lower conductivity (145.5 µS/cm), turbidity (3392.5 NTU), colour (3375 Hz), and TSS (3630 mg/L). At Buabenso, the average pH was 6.98, with conductivity of 146.75 µS/cm, turbidity of 3525 NTU, colour of 3812.5 Hz, and total suspended solids (TSS) of 3857.5 mg/L. Kyekyewere showed a slightly lower pH of 6.95 and the lowest conductivity (145.25 µS/cm), but recorded the highest turbidity (3725 NTU), colour (4175 Hz), and TSS (4342.5 mg/L) among the three sites.

**Table 2:** Laboratory-based water quality parameters

Parameter	Adwuman	Buabenso	Kyekyewere
pH	6.98	6.98	6.95
Conductivity (µS/cm)	145.5	146.75	145.25
Turbidity (NTU)	3392.5	3525	3725
Colour (Hz)	3375	3812.5	4175
TSS (mg/L)	3630	3857.5	4342.5

### Temporal land cover changes across the three communities

**Fig. 2:**
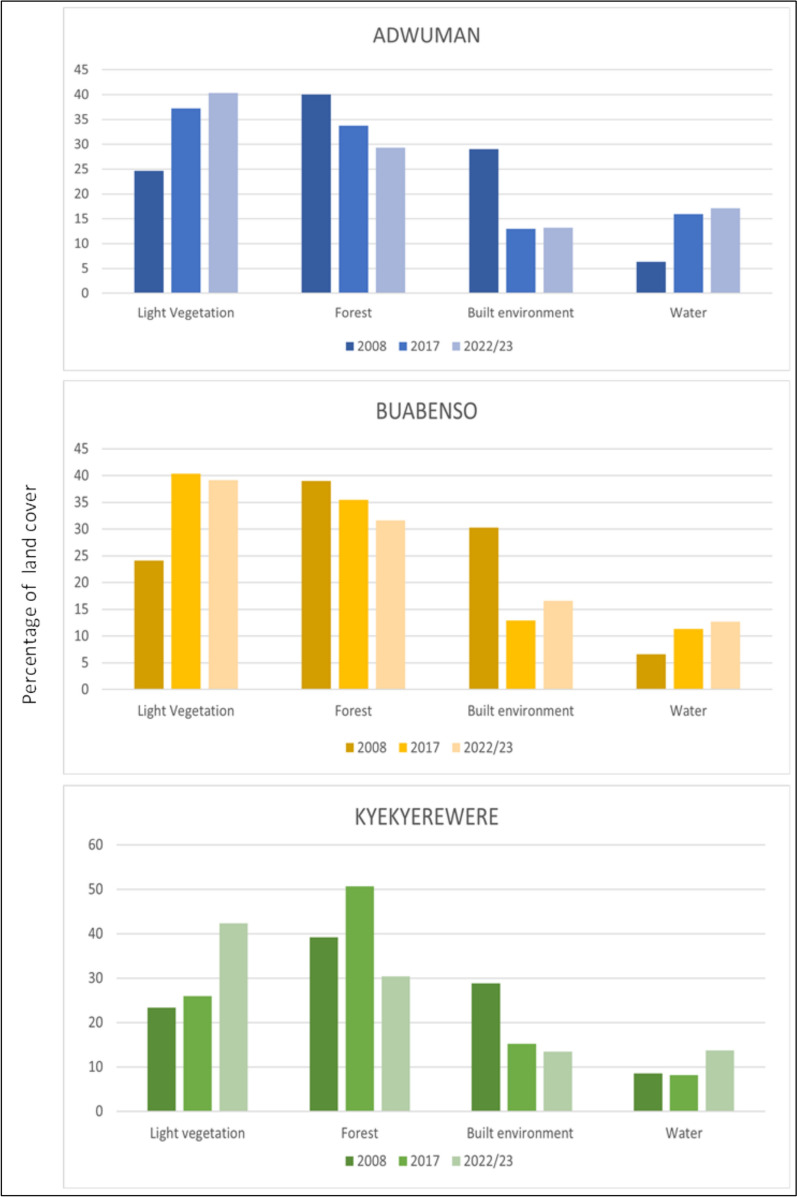
A clustered bar graph showing the comparative landcover transitions across Adwuman, Buabenso and Kyekyewere

Land cover analysis for Adwuman from 2008 to 2022/23 showed notable shifts in the landscape composition. Light vegetation increased from 24.64% in 2008 to 37.26% in 2017, and 40.35% in 2022/23, representing a total rise of 15.71 percentage points (Fig. 2). Forest cover declined, from 40.03% in 2008 to 33.78% in 2017 and 29.31% in 2022/23, a total decrease of 10.72 percentage points. The built environment decreased sharply between 2008 and 2017, from 29.03 to 13.02%, and remained stable at 13.21% in 2022/23. Water coverage increased, from 6.31% in 2008 to 15.94% in 2017 and 17.12% in 2022/23, a total rise of 10.81 percentage points.

Temporal analysis of land cover change in Buabenso from 2008 to 2022/23 showed notable shifts in landscape composition (Fig. 2). Light vegetation increased from 24.12% in 2008 to 40.33% in 2017, a rise of 16.21 percentage points, before slightly declining to 39.12% in 2022/23, given a net increase of 15.00 percentage points over the study period. Forest cover declined from 39.01% in 2008 to 35.44% in 2017 and 31.60% in 2022/23, a total reduction of 7.41 percentage points. The built environment decreased from 30.27% in 2008 to 12.88% in 2017, then increased to 16.56% in 2022/23, resulting in an overall reduction of 13.71 percentage points. Water coverage increased from 6.60% in 2008 to 11.35% in 2017 and to 12.72% in 2022/23, a total increase of 6.12 percentage points.

Temporal analysis of land cover change in Kyekyewere from 2008 to 2022/23 showed pronounced shifts in landscape composition (Fig. 2). Light vegetation increased from 23.41% in 2008 to 25.98% in 2017, a rise of 2.57 percentage points, and then rose sharply to 42.34% in 2022/23, adding a further 16.36 percentage points for a total increase of 18.93 percentage points. Forest cover increased from 39.22% in 2008 to 50.65% in 2017 a rise of 11.43 percentage points, before declining to 30.42% in 2022/23, a reduction of 20.23 percentage points, resulting in a net decrease of 8.80 percentage points. The built environment decreased from 28.87% in 2008 to 15.19% in 2017, a reduction of 13.68 percentage points, and further declined to 13.46% in 2022/23, giving a total reduction of 15.41 percentage points. Water coverage remained relatively stable between 2008 (8.51%) and 2017 (8.18%), a decrease of 0.33 percentage points, before increasing to 13.77% in 2022/23, a rise of 5.59 percentage points, resulting in a net gain of 5.26 percentage points.

### Spatial expression of land cover change from ISODATA/PRC outputs

**Fig. 3:**
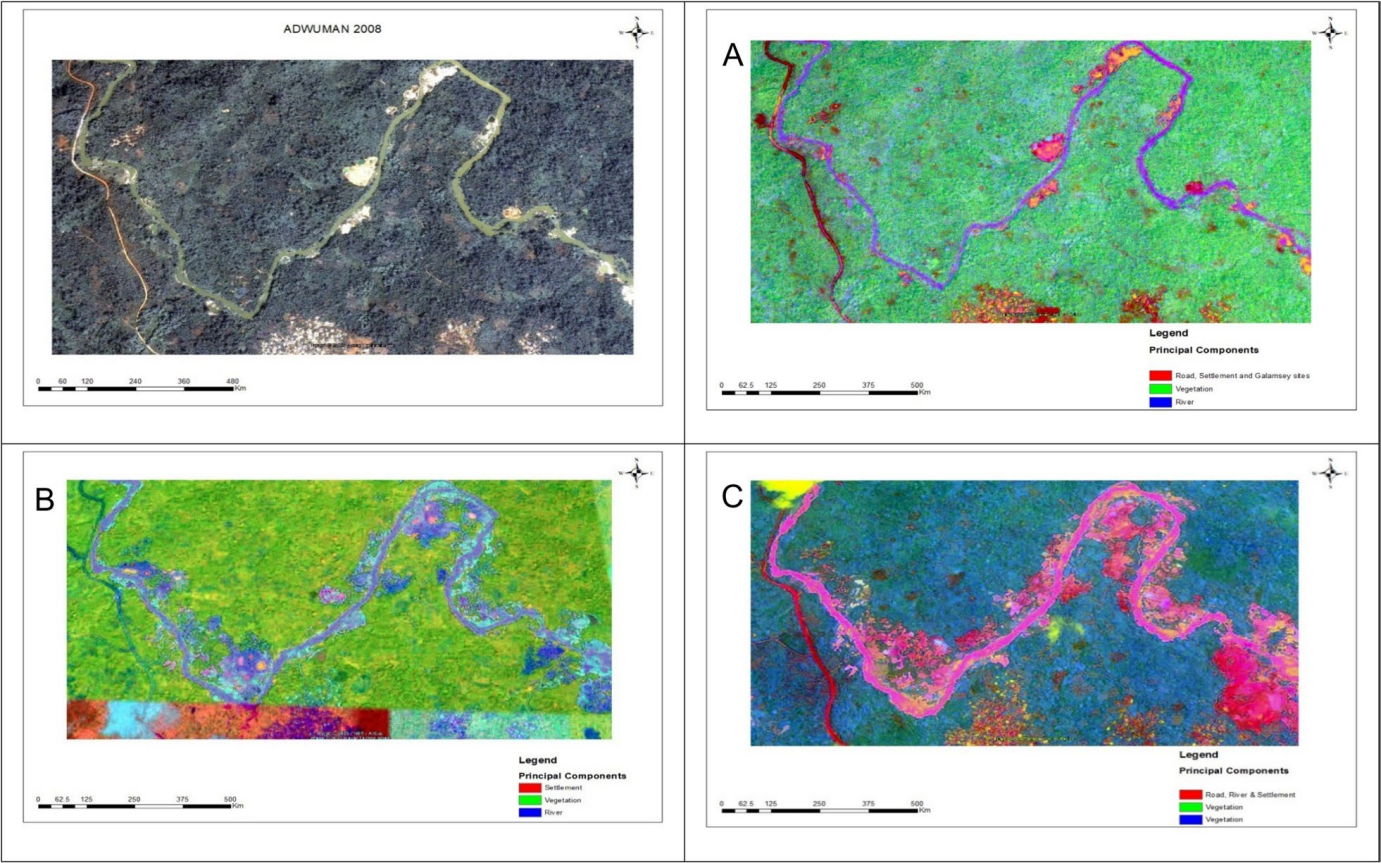
Satellite imagery of Aduman (2008) and Principal Raster Component outputs of land cover change (**A**-2008, **B**-2017, **C**-2023)

The ISODATA/PRC composites for Adwuman showed progressive spatial changes in land cover between 2008, 2017 and 2022/23 (Fig. 3). The component patterns distinguished major land cover classes, with spectral variation reflecting differences among vegetation, built-up areas and water bodies. The composites showed consistent spatial patterns, including increases in light vegetation and water features and deductions in forest cover and built-up areas. In 2008, the composites showed limited areas of disturbed land. By 2017, composites indicated river expansion and increased areas of fragmented vegetation. The 2022/23 composite showed additional river widening and extensive modification of adjacent land consistent with spatial trends observed in earlier years.

**Fig. 4:**
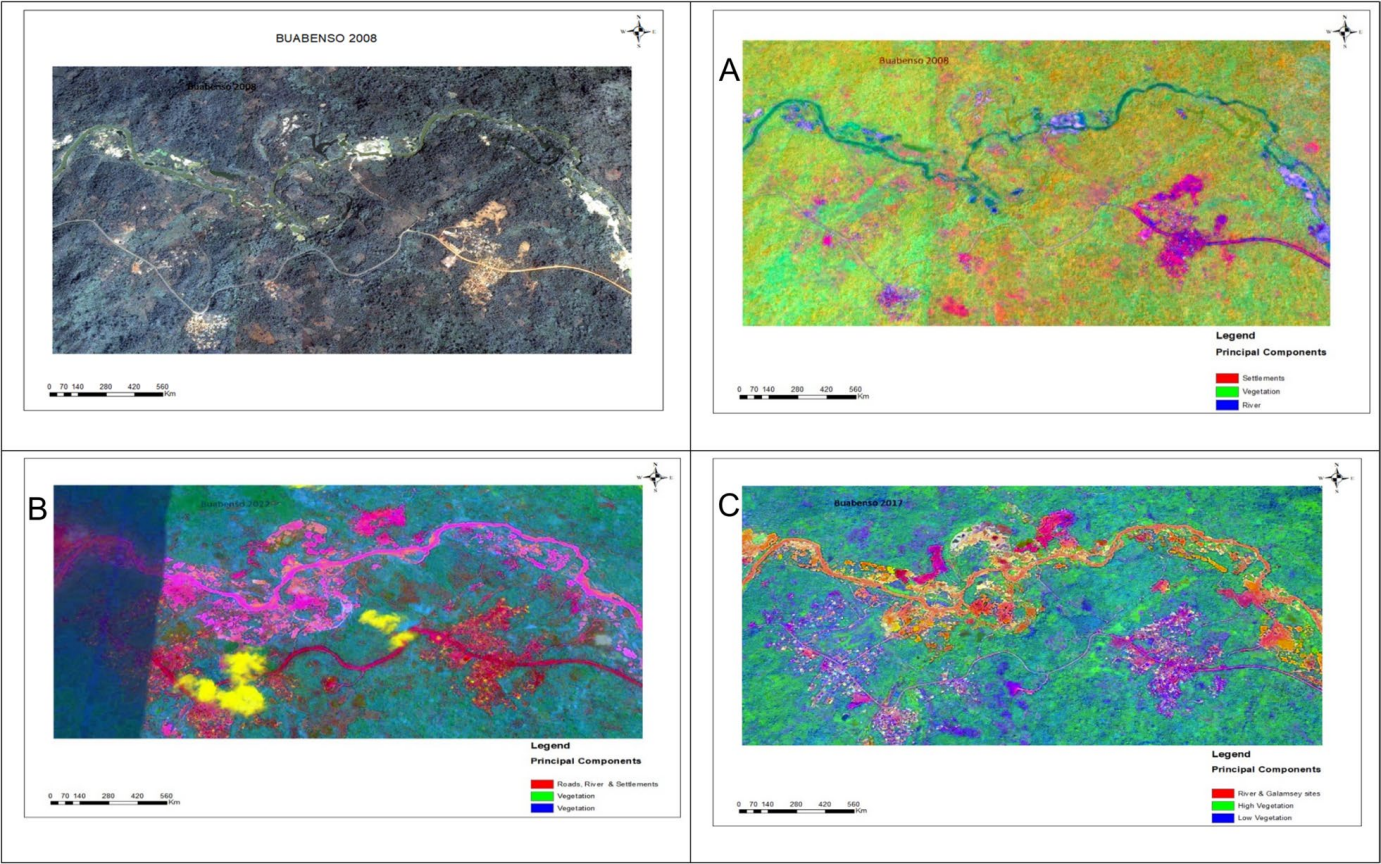
Satellite imagery of Buabenso (2008) and Principal Raster Component outputs of land cover change (**A**-2008, **B**-2017, **C**-2023)

The ISODATA/PRC composites for Buabenso showed progressive spatial changes in land cover from 2008, 2017, to 2022/23 (Fig. 4). The composite patterns distinguished major landcover classes, reflecting spectral differences among vegetation, built-up areas and water bodies. The composites showed increasing light vegetation over time, accompanied by gradual forest loss and fluctuations in built-up areas. In 2008, composites showed a relatively balanced distribution of vegetation and built-up areas. By 2017, the spatial indicated substantial expansion of light vegetation and a marked decline in built-up cover. The 2022/23 composite showed continued forest decline, moderate increases in developed areas and further expansion of water-dominated pixels. These spatial patterns were consistent with the classified land-cover outputs, indicating substantial landscape modification over the study period.

**Fig. 5:**
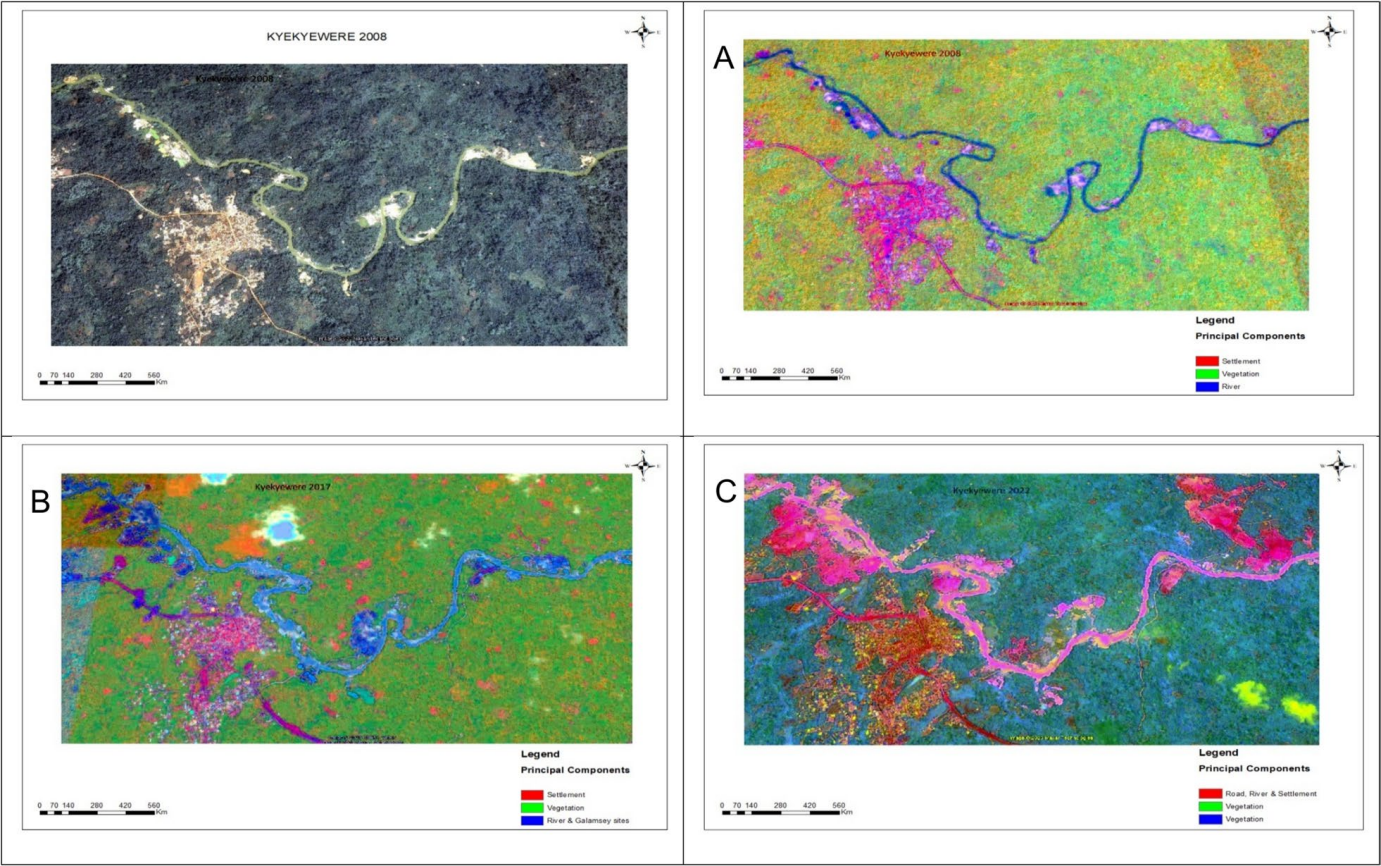
Satellite imagery of Kyekyewere (2008) and Principal Raster Component outputs of land cover change (**A**-2008, **B**-2017, **C**-2023)

The ISODATA/PRC composites for Kyekyewere showed distinct spatial transformations between 2008, 2017 and 2022/23 (Fig. 5). The component patterns differentiated major land cover classes, reflecting spectral variation across vegetation, built-up areas and water bodies. The composites showed a modest increase in light vegetation between 2008 and 2017, followed by a substantial expansion by 2022/23. Forest cover increased initially, but declined sharply by 2022/23, while built-up areas showed a consistent reduction across the study period. Water features remained relatively stable between 2008 and 2017, with a notable increase in 2022/23. These spatial patterns were consistent with the classified land cover and showed substantial reconfiguration of land cover distribution over time.

### NDVI-based vegetation health assessment

**Fig. 6:**
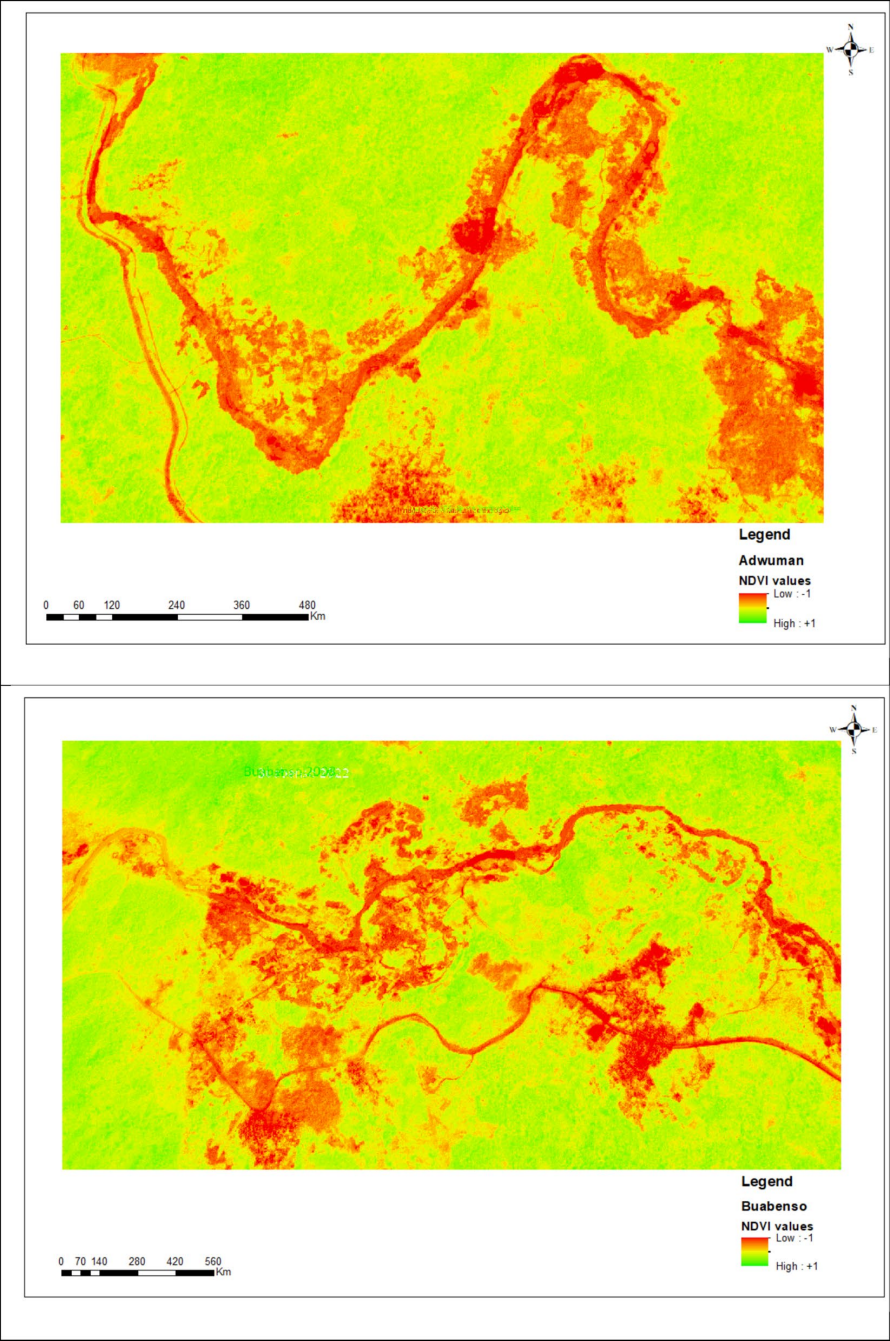

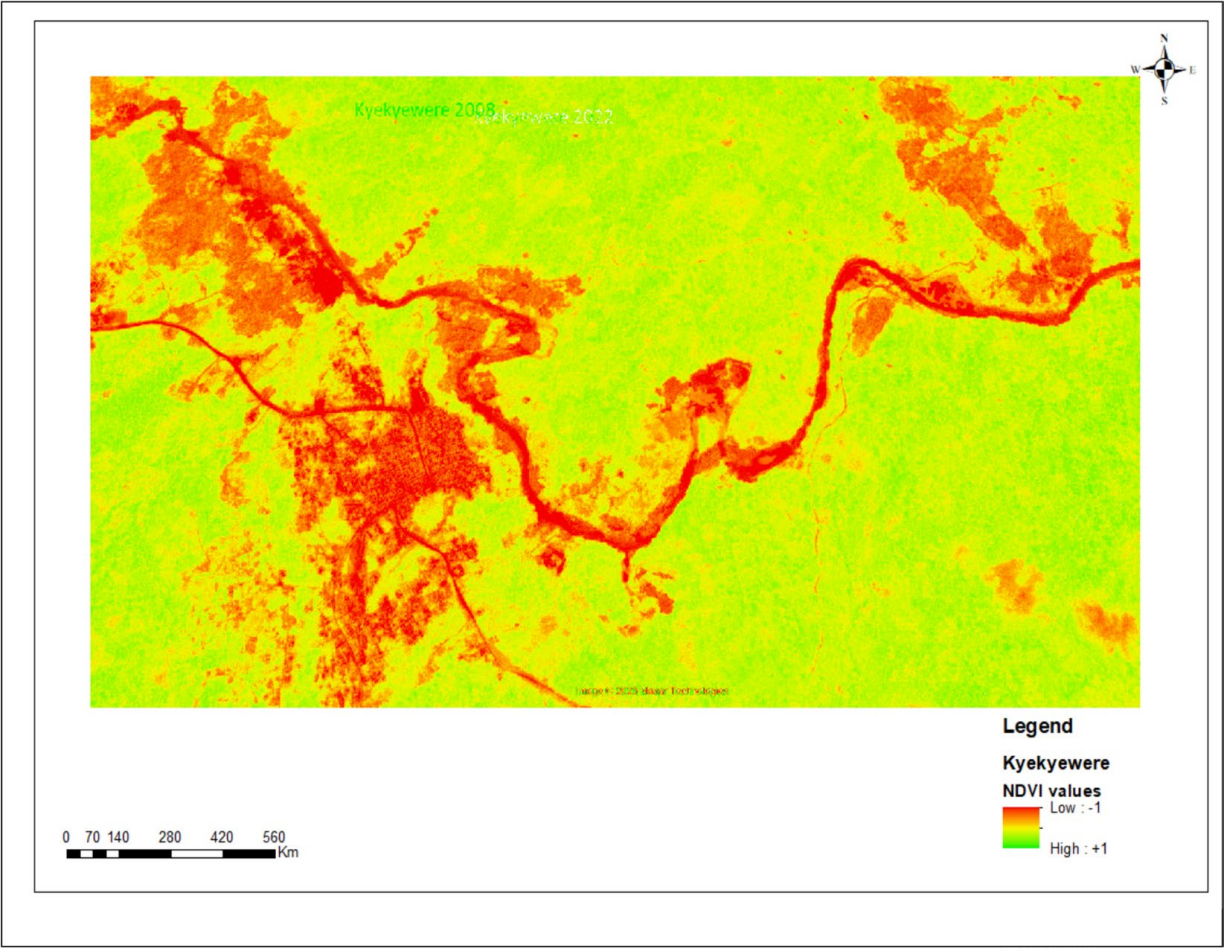
NDVI showing areas of disrupted vegetation in the 3 communities

NDVI analysis revealed spatiotemporal variation in vegetation health across Adwuman, Buabenso, and Kyekyewere between 2008 and 2022/23 (Fig. 6). All three communities showed a decline in areas with high NDVI values, indicating reduced coverage of dense healthy vegetation. There was a corresponding expansion of areas classified as sparse or moderately stressed vegetation, particularly near riverbanks. Kyekyewere showed the most pronounced increase in low NDVI zones in the 2022/23 imagery, while Buabenso and Adwuman exhibited moderate but consistent reductions in vegetative vigor over time. These NDVI patterns were consistent with the land cover classification results, showing progressive changes in vegetation condition across the study sites.

## Discussion

This study was initiated in response to reports of declining blackfly breeding populations along the Ofin River and tributaries in Ghana, particularly in communities under routine surveillance by the Neglected Tropical Disease Elimination Program. The reported gradual absence of these vectors by the programme and previous studies [15, 16], which are essential to the transmission of *Onchocerca volvulus*, raised concerns about broader environmental disruptions potentially linked to unregulated mining activities [17, 25]. The research therefore assessed ecological conditions across three riverine communities (Adwuman, Buabenso, and Kyekyewere) by examining water quality, land cover change and vegetation health where illegal mining activities are ongoing.

Hydrological assessments showed that river discharge and pH remained within ranges typically associated with suitable blackly larval habitats as corroborated by Onifade et al. [26]. Temperature, conductivity and total dissolved solids also fell within reported ecological tolerance for blackflies that notwithstanding, the physical quality of the water had deteriorated markedly. All three sites recorded turbidity exceeding 3300 NTU, far above levels associated with viable blackfly habitats [27] with Kyekyewere showing the most extreme elevated values. A similar study along the Ofin and Pra rivers have similarly reported high turbidity associated with mining activity and concurrent reductions in blackfly populations especially in the dry season [17]. Total suspended solids were also elevated (3630–4342 mg/L) and colour values (3375–4175 Hz) indicated high levels of suspended particulates [28]. These conditions are known to impair larval attachment and respiration, as blackfly larvae require clean and fast-flowing water with stable substrates [29]. The excessive sedimentation observed may smother potential breeding surfaces and reduce habitat suitability for blackfly development [30]. Taken together, the high sediment load suggests ecological conditions that are less favourable for blackfly breeding, consistent with patterns reported in other mining-affected regions. Findings from previous entomological studies support the interpretation that blackfly population declines in mining-affected areas may reflect broader ecological degradation, rather than isolated entomological anomalies [31, 32].

The spatial analysis using Principal Raster Components (PRC) revealed consistent patterns of land cover transformation across the study sites. All three communities showed reductions in forest cover accompanied by increases in light vegetation and disturbed surface areas. These shifts indicate deforestation, riparian fragmentation and expansion of disturbed landscapes, changes that reduce the shaded, vegetated microhabitats typically associated with blackfly breeding [32]. In Kyekyewere, landscape change was particularly pronounced, with an initial period of forest cover followed by rapid degradation by 2023. This volatility is consistent with the episodic and land disturbance commonly associated with illegal mining activities [33].

The NDVI analysis further confirmed a decline in vegetative health across all three communities. Areas previously characterized by dense, photosynthetically active vegetation have transitioned into zones of sparse or stressed cover. These changes were especially pronounced near riverbanks and known galamsey sites, suggesting a spatial association between mining activity and localized ecological decline [34, 35]. The reduction in NDVI across the study periods aligns with our PRC-derived land-cover transitions and indicates that vegetation loss is both widespread and ongoing.

Published entomological data from nearby regions provided useful comparative insight into how ecological changes associated with mining may influence blackfly populations. In the Upper Denkyira District (our study district), long-term assessments from 2001 to 2014 documented a substantial decline in *Simulium* populations. Early collections reported several thousand flies with moderate parous rates but by 2006 the numbers had declined significantly [18]. By 2013/2014, *Simulium* populations had largely disappeared from monitored rivers, with only small numbers detected in unmined tributary [17]. Similar patterns were observed, in Bosomase along the Pra River, downstream of our study communities, where initially high fly numbers were followed by marked fluctuations over subsequent years. After a brief period of elevated activity, populations collapsed sharply subsequently [16]. These declines have been associated in previous studies with intensified mining activity, suggesting a possible link between ecological disruption and reduced vector population.

Beyond potential impacts on blackfly habitats, the ecological consequences of illegal mining extend to broader community well-being and sustainable development. The degradation of vegetative cover and deterioration of water quality compromises agricultural productivity and threaten food security for subsistence farmers who rely on fertile land and clean water for their livelihoods [36]. In several regions of Ghana, illegal mining has already displaced farming communities, reduced crop yields, and undermined household resilience [37]. These impacts intersect with several Sustainable Development Goals (SDGs) including SDG 2 (Food security), SDG 3 (Good Health), SDG 6 (Clean Water), and SDG 15 (Ecosystem protection). Mining-related land degradation, declining water quality, and biodiversity loss can erode ecological integrity and contribute to increased poverty, health disparities, and social vulnerability [38–41].

The study had some limitations. First, only a limited set of water quality parameters was measured, and key pollutants such as heavy metals and organic contaminants were not assessed. Including these parameters would have provided a more comprehensive assessment of river contamination [17]. Also, although previous studies and by the NTD programme reports indicated low blackfly densities in the region, the study did not include entomological sampling and seasonal variations may influence reported densities [42]. Therefore, any link between ecological degradation and reduced blackfly populations should be interpreted cautiously and supported by future entomological monitoring. Further, the PRC‑derived land cover classifications showed a reduction in built‑up area, which contrasts with observed community expansion and mining activity. This discrepancy likely reflects spectral confusion between built‑up surfaces and bare ground, as well as the limitations of unsupervised PRC analysis in capturing heterogeneous or informal structures.

## Conclusion

The findings of this study indicate that illegal mining activities have contributed to substantial ecological changes along the Ofin River and its surrounding communities. The combined effects of extreme sedimentation, vegetation loss, and riparian disturbance suggest conditions are less favourable for blackfly breeding aligning with patterns reported in other mining affected regions. These environmental changes reflect a broader pattern of ecological degradation with implications for public health, food security, and sustainable development. Addressing the impacts of galamsey (illegal mining) will require environmental restoration, strengthened community resilience, and the protection of riverine ecosystems essential for both ecological and human health.

## Supplementary Information

Supplementary material 1.

## Data Availability

All data used for the manuscript has been included in the paper. Further request can be made to the corresponding Author.

## Abbreviations

*Sl*: Sensu lato
PC: Preventive chemotherapy
NTD: Neglected tropical diseases
ASM: Artisanal and small-scale mining
SDGs: Sustainable development goals
TDS: Total dissolved solids
CSIR: Council for Scientific and Industrial Research
PRC: Principal Raster components
NDVI: Normalized difference vegetation index
HLC: Human landing catches
MBRs: Monthly biting rates
MPBRs: Monthly porous biting rates
TSS: Total suspended solids
