# Supplementary material for: A GIS and field-based assessment of the ecological consequences of illegal mining (galamsey) on blackfly breeding sites in Ghana: implications for the sustainable development goals
Source: Trop Med Health. 2026 Mar 31;54:74. doi: 10.1186/s41182-026-00944-4 (PMC13104455; doi:10.1186/s41182-026-00944-4)
Supplement: Supplementary file 1 — Supplementary material 1. [file 41182_2026_944_MOESM1_ESM.docx]

| 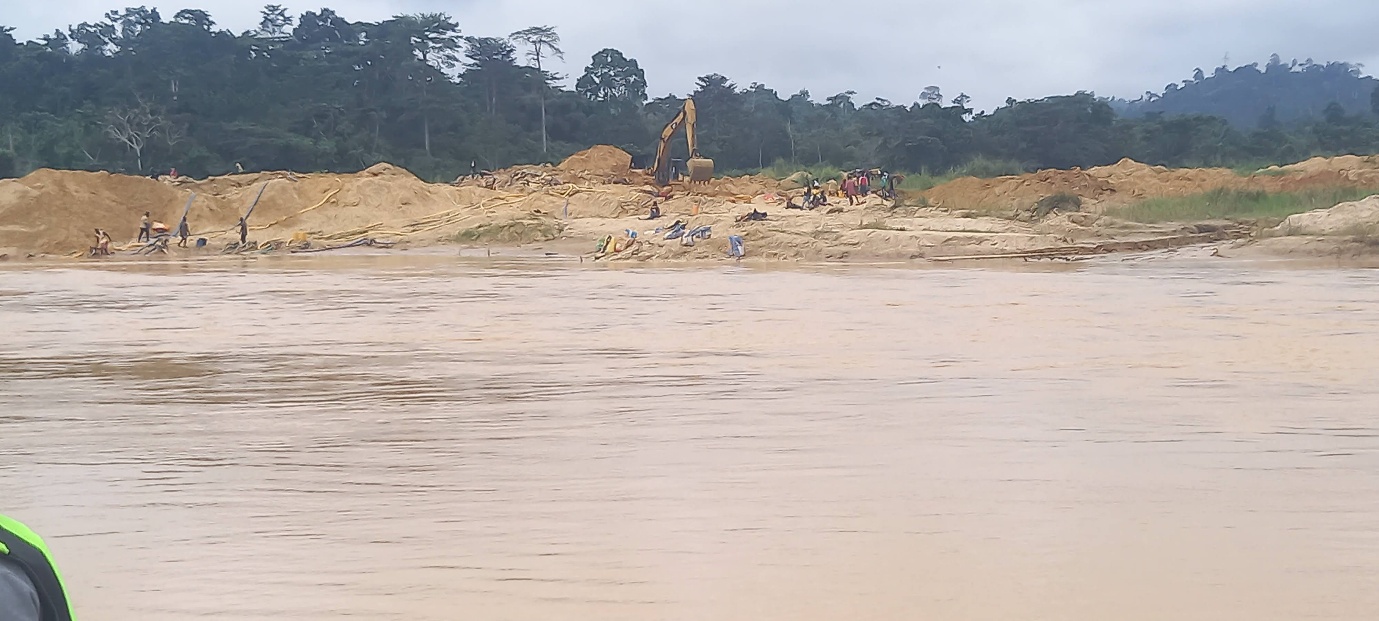 |
| --- |
| 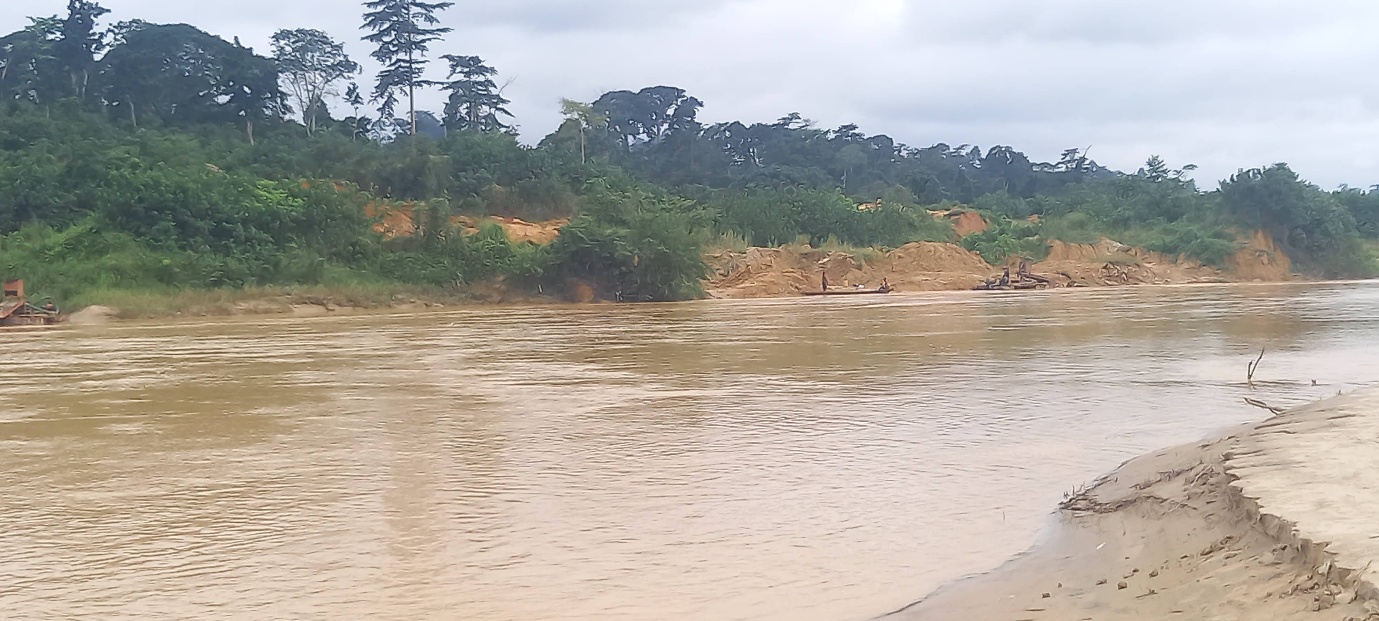 |
| 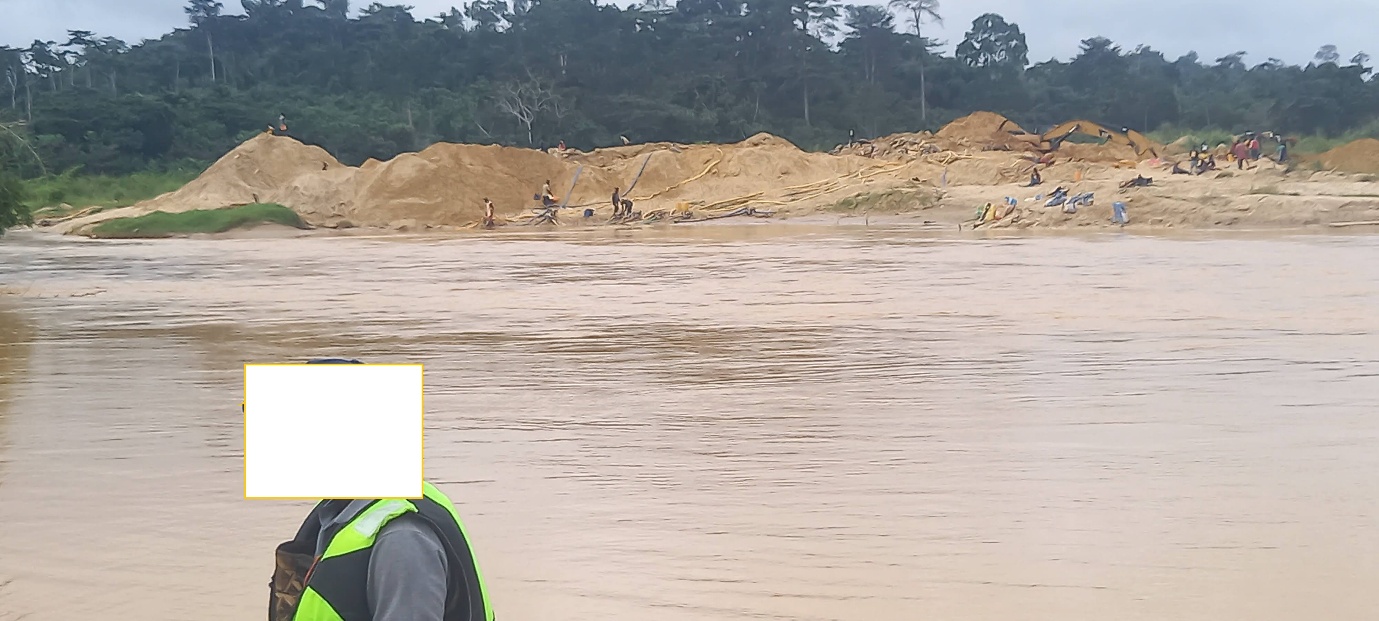 |

Supplementary Figure S1. Field photographs showing active small‑scale mining along the Ofin River during water‑quality and flow measurements. Visible disturbance to riverbanks and adjacent vegetation illustrates the ongoing environmental impact of mining activities in the study area.
